# Clarifying Mutual Consent’s Role in Agency Law

**DOI:** 10.1093/ojls/gqae031

**Published:** 2024-09-02

**Authors:** Rachel Leow

**Affiliations:** Associate Professor, London School of Economics and Political Science

**Keywords:** agency law, mutual consent, commercial law, powers, duties

## Abstract

Important cases and academic commentators have suggested that the mutual consent of principal and agent is necessary for actual authority to be conferred on the agent. The chief purpose of this article is to show that this view of mutual consent’s role in agency law is inaccurate and misleading. Its central claim is that the agent’s consent is not a necessary pre-condition for the conferral of authority. Instead, a principal can confer authority on an agent unilaterally. However, when authority is conferred unilaterally on an agent, the external aspect of agency is fully present, but the internal principal–agent relationship possesses two unique features, one relating to the agent’s duties and the other relating to the agent’s ability to disclaim. The account presented here thus clarifies the proper scope of ‘mutual consent’ justifications in agency. Mutual consent may justify some incidents of agency, but it does not justify them all.

## 1. Introduction

Over several decades, courts^1^ and important writers^2^ have suggested that the mutual consent of principal and agent is at the heart of agency. Such statements have been extremely persuasive, leading many to regard mutual consent as the key normative justification for agency. But agency is a broad field. Even proponents of mutual consent accounts typically accept that mutual consent cannot explain or justify some areas of agency law, such as apparent authority or agency of necessity. Statements about the centrality of mutual consent to agency thus require further unpacking and clarification.

One possibility is that such statements are claims about what is arguably agency’s core concept: the conferral (or grant)^3^ of actual authority by the principal on the agent.^4^ On this view, the mutual consent of principal and agent is a necessary condition for authority to be conferred on the agent (though no contract between the two is required). Authority is conferred if and only if the principal manifests his consent to the agent acting for him, and the agent likewise manifests his consent to so act for the principal.^5^ This view is adopted by some prominent cases and academic commentators, and implicitly assumed by others, though sometimes imperfectly.

The chief purpose of this article is to show that this view of mutual consent’s role in agency law is inaccurate and misleading, thereby clarifying the role of mutual consent in agency law. Its central claim is that, contrary to some important views, mutual consent is not a necessary condition for the conferral of authority.

The article proceeds in three steps. First, it shows that a principal can confer authority on an agent unilaterally, without the agent’s consent or even his knowledge. Thus, mutual consent is not a necessary condition for the conferral of authority. Five pieces of evidence demonstrate this: powers of attorney; the case of *Ruggles v American Central Insurance Company*;^6^ the law on the revocability of authority; ratification; and other cases on the conferral of powers more generally. Although the existence of some of this evidence has long been recognised, its significance has not been sufficiently appreciated.

Second, the article explains the nature of the relationship created if a principal confers authority on an agent unilaterally and why such conferral is unobjectionable. When authority is conferred unilaterally on the agent, the agent is empowered to affect the principal’s legal relations with third parties within the scope of that authority (the external aspect of agency), but the principal’s legal relations with the agent (the internal aspect of agency) possess two exceptional features. The first is that the agent will owe no agency-specific duties to the principal until and unless he consents to act for the principal. The second is that, only in these cases, the agent should have the option of disclaiming the authority. Disclaimer has retrospective effect, so that, having disclaimed, the agent is treated as if authority had never been validly conferred. These features avoid two objections that might otherwise be cast at the unilateral conferral of authority: first, that the imposition of agency-specific duties would be unduly onerous for the agent; and second, that no one should be compelled to be an agent against their will.

Third, the article shows how the account presented here casts doubt on the accepted wisdom, present to various extents in virtually every major account of agency, that mutual consent is at the heart of agency. At least in relation to the conferral of authority, such statements are untrue. At the heart of the conferral of authority is a unilateral act by the principal. However, statements about mutual consent’s centrality to agency may not need to be rejected in full if more effort is taken to carefully distinguish between agency’s different incidents and the reasons for their creation. Mutual consent is a reason why some incidents arise but not others. Such statements may still be salvaged if we are more precise about exactly what we mean by ‘agency’.

Thus, the aim of this article is not to call for any radical change of the law, but to better understand the law that we already have. In doing so, the article makes three key contributions: (i) it shows that the principal can unilaterally confer authority on an agent; (ii) it explains the features of the relationship thereby created when the principal does so; and (iii) it clarifies the proper scope of ‘mutual consent’ justifications in agency.

## 2. ‘Mutual Consent is at the Heart of Agency’

Statements that the mutual consent of the principal and agent is at the heart of agency abound. A classic example is provided by *Bowstead & Reynolds*, the leading English text on agency, which starts its definition of agency as follows:

Agency is the fiduciary relationship which exists between two persons, *one of whom expressly or impliedly manifests assent* that the other should act on his behalf so as to affect his legal relations with third parties, and the other of whom *similarly manifests assent so to act* or so acts pursuant to the manifestation.^7^

Across the Atlantic, Professor Seavey, the first Reporter of the highly influential US *Restatement on Agency*, once defined agency in similarly consensual terms. Agency is ‘a consensual relationship in which one (the agent) holds in trust for and subject to the control of another (the principal) a power to affect certain legal relations of the other’.^8^ Professor Seavey’s definition led him to reject the idea of ‘agency created by law’ since such agency relationships would not be consensually created.^9^ In his view, those cases simply fall outside the definition of agency.

Seavey’s influence is still evident over 100 years later. Today, the *Restatement (Third) of Agency* still maintains a similar line, explaining that:

Agency is the fiduciary relationship that arises when one person (a ‘principal’) manifests assent to another person (an ‘agent’) that the agent shall act on the principal’s behalf and subject to the principal’s control, and the agent manifests assent or otherwise consents so to act.^10^

In these important works on agency, the mutual consent of principal and agent is regarded as so important that they form part of the definition of agency itself.

Such statements, however, are often qualified. For example, Professor Munday recognises that ‘Many would claim that consent lies at the heart of agency’,^11^ but simultaneously accepts that this claim cannot accommodate all aspects of agency law. Some qualifications are extremely well rehearsed: for example, that mutual consent of the principal and agent is unnecessary for apparent authority to exist, or that agency of necessity (if it exists) is an exception.^12^ However, such exceptions are often downplayed as controversial outliers or otherwise explained away in more-or-less consensual terms.^13^ The way that these limited exceptions are treated thus buttresses the centrality of mutual consent to agency law.^14^

It thus seems uncontroversial that mutual consent is at the heart of agency law. But agency law is a broad area. What might this claim be referring to?

One possibility is that the claim concerns a phenomenon at the core of agency: the conferral (or grant) of actual authority by the principal on the agent.^15^

The conferral of authority is one aspect of the internal relationship between principal and agent. However, it is conceptually separate from any contract which may exist between the two. Authority can be conferred even if no contract exists, for example because of the principal’s or the agent’s minority and hence lack of contractual capacity.^16^ Conversely, the conferral of authority can be revoked by the principal even if doing so puts the principal in breach of a contract with the agent.^17^

Of the two aspects of the internal relationship, the conferral of authority is arguably the key concept. It describes the process and result by which an agent is invested with the ability^18^ to act for the principal in altering the principal’s legal relations with third parties, which is generally regarded as the characteristic feature of agency.^19^

## 3. Is Agent Consent a Necessary Pre-Condition for the Conferral of Authority?

Claims that mutual consent is at the heart of agency have sometimes been understood as claims about the conferral of authority. On this view, the mutual consent of principal and agent is a necessary condition precedent for actual authority to be conferred by the principal on the agent. I present this account and contrast it with an opposing one. I also show how the leading English agency text, *Bowstead & Reynolds*, is ambiguous about which account is adopted. I then explain why which approach is taken has important practical implications.

### A. Pre-condition to the Conferral of Authority

Some important writers and cases clearly regard the agent’s consent as a necessary pre-condition for the conferral of authority.

As Professor Howard Bennett states:

For actual authority to be conferred, there must be a manifestation of the voluntary grant of authority by the principal to the agent, and the agent must manifest its consent to assume the role of agent within the terms of the grant of authority.^20^

Likewise, Professor Dal Pont describes ‘the consent (or assent) of both principal and agent’ as an ‘essential’ element of agency.^21^ In his view, ‘Consent (or assent) requires some acceptance by the agent of a mandate, whether or not formal or contractual, from the principal; agency is usually not something that can be “unilaterally thrust upon a prospective agent”’.^22^

Similar views are found in the case law. In the House of Lords case of *Garnac Grain Co v Faure & Fairclough Ltd*, Lord Pearson held that:

The relationship of principal and agent can only be established by the consent of the principal and the agent. They will be held to have consented if they have agreed to what amounts in law to such a relationship, even if they do not recognise it themselves and even if they have professed to disclaim it … But the consent must have been given by each of them, either expressly or by implication from their words and conduct.^23^

These observations in *Garnac Grain* have been followed by numerous subsequent cases in England and Wales,^24^ and even in Scotland.^25^

Elsewhere in the Commonwealth, the Federal Court of Australia has said that ‘In general, no formality is necessary for the appointment of an agent to act on behalf of his principal … It is only necessary that the principal and agent consent to the relationship.’^26^ Similarly, the Singaporean Court of Appeal in *Alwie Handoyo v Tjong Very Sumito* said that ‘Clearly and self-evidently, the agent itself needs to consent to being the agent’^27^ and ‘Consent by the agent is indisputably required to form an agency relationship’.^28^

These statements emphasise that the agent’s consent is a necessary pre-condition for the relationship of principal and agent to arise. That relationship, in turn, is generally constituted by the conferral of authority by the principal on the agent.^29^ They thus support the view that the agent’s consent is necessary for authority to be conferred on him.

### B. Not a Pre-condition to the Conferral of Authority

Not all agree with the view just outlined. A contrary view is that, while the principal’s consent to the agent acting for him is necessary for the conferral of authority, the agent’s consent is not. This view was clearly adopted by Professor Wolfram Müller-Freienfels in his scholarly comparison of agency principles between civilian and common law jurisdictions. He observed that, in the common law:

Unlike the contract which is necessarily a bilateral manifestation of assent, there is no conceptual reason why the grant of agency power should not be construed as a unilateral manifestation to the agent by the principal alone; agreement as well as consideration may be lacking. Neither is there a need that the agent, or the third party, should have received notice of this act. *The agent acquires authority even if he does not consent to the authorization of the principal vis-à-vis the principal*.^30^

This suggests that the agent’s consent is not a necessary pre-condition for the conferral of authority.

### C. Bowstead & Reynolds

Some other writers adopt a position that is more difficult to pin down. Consider in particular *Bowstead & Reynolds*, which seems to adopt both positions at different points in the book.

*Bowstead & Reynolds* makes it clear that the agency relationship may be constituted *inter alia* ‘by the conferring of authority by the principal on the agent’.^31^ The editors go on to explain in the next paragraph that

the essence of agency must lie in a unilateral manifestation of will, but that very often arises from … a contract between principal and agent … These features make it appropriate to attribute in exposition a fundamental role to consensual agency.^32^

These statements appear ambiguous as to whether the agent’s consent is a necessary condition for the conferral of authority. The first phrase suggests that it is not, since the ‘essence’ of agency lies in a unilateral manifestation from the principal, but the last sentence suggests that it is.

This ambiguity is present at other points in *Bowstead & Reynolds*. Although, as discussed earlier, *Bowstead & Reynolds* initially defines agency in terms which seem to require both the principal and agent’s consent to act, the editors later say that: ‘The basic justification for the agent’s power as so far explained seems to be the idea of a unilateral manifestation by the principal or willingness to have his legal position changed by the agent.’^33^

This latter view is reiterated as follows:

It is traditional to state that the agent’s assent (or consent) is required, and to discuss the ways in which this can be implied from acts or waived by the principal … But as regards the position between principal and third party, the relevant act is the conferring of authority. It is suggested above that the basis of agency is a unilateral manifestation of will: a power of attorney, for instance, does not require acceptance by the donee of a power.^34^

Again, this presents an apparent inconsistency, perhaps even a contradiction. While the definition of agency suggests that the agent’s consent is necessary for an agency relationship to be constituted, most commonly by the conferral of authority, the later quotes suggest that authority can be conferred even where the agent does not consent to act for the principal.

The recent High Court decision of *Libyan Investment Authority v King* relied on *Bowstead & Reynolds* in producing the following summary of principles:

iii) The relationship of principal and agent can be constituted by the conferral of authority by the principal on the agent, which may be express or implied from conduct. The conferral of authority is voluntary or consensual but a formal contract is not required.

iv) A unilateral manifestation of will by the principal is important since this is the basic justification for the agent’s power.

v) It is sufficient if the principal manifests to the agent that the principal is willing to have its legal position changed by the agent.

…

vii) Both principal and agent must assent to the agency. Where mutual assent is to be implied, the correct test is whether one party conducted itself towards the other in such a way that it was reasonable for that other to infer assent.^35^

Reflecting the ambiguity in *Bowstead & Reynolds*, these statements are internally inconsistent. While principles (iv) and (v) suggest that actual authority can be conferred unilaterally by the principal, principle (vii) indicates the contrary position.

Similar inconsistencies can be found elsewhere. One of *Bowstead & Reynolds*’s editors, Professor Francis Reynolds, has recently said elsewhere that ‘Although a unilateral act by the principal, the conferral [of authority] probably also requires some sort of acceptance by the agent’.^36^ The first part of the sentence suggests that the agent’s consent is not necessary, but the latter part suggests that it is.

Given these inconsistencies, there is value in looking at the position afresh. This is especially so since which view is adopted has important practical implications.

### D. Practical Implications

Whether the agent’s consent is a necessary pre-condition for the conferral of authority can be practically important, affecting the outcome of cases.

Consider cases where P gives A authority, which A is unaware of. For example:

*Overseas Agent*: P executes a power of attorney conferring power on A to buy goods for P. P sends the power of attorney deed to A’s registered address. Unknown to P, A has moved overseas and does not know of the existence of the power of attorney.

Whether A has authority to buy goods for P turns on whether A’s consent to act as agent is necessary for authority to be conferred on A. If A’s consent is necessary, then A will have no authority in this case, as A has not consented to act for P. If A then purports to exercise the authority by buying goods for P from a third party T, then P and T will not owe contractual obligations to each other.

However, if A’s consent is not necessary for the conferral of authority on A, the result will be the opposite: A will have authority and P and T will be contractually bound to each other. If T fails to deliver the goods, P can sue for breach of contract; if P fails to pay T, T can sue for the price.

Similar situations may arise for many reasons. Intended communications by P to A may be lost, or they may be delayed because of a postal strike. P may confer wider authority on A but inform A that he has only a narrower authority by mistake, or P may first give A a narrower authority and then confer on A a wider authority in writing which A does not receive. In all these cases, the outcome depends on whether the agent’s consent is a necessary pre-condition for authority to be conferred.

Similarly, P may give A authority to passively receive notifications, such as service of process^37^ or notices to quit:^38^

*Notices*: P, a landlord, executes a written authority conferring power on A to receive notifications from his tenants and sends the written authority to A on 1 January by post. The post is delayed, and the written authority only reaches A on 31 January. On 5 January, one of P’s tenants gives A notice to quit by leaving a note on A’s front door.

Again, whether the notice to quit has been validly given on 5 January depends on whether the agent’s consent is necessary for authority to be conferred on the agent.

## 4. Agent Consent Not a Necessary Condition for the Conferral of Authority

In this section, I argue that the second view is right: the agent’s consent is not a necessary condition for authority to be conferred on him. Instead, authority can be conferred by the principal unilaterally. Five sources of evidence can be marshalled in its favour: powers of attorney; the case of *Ruggles v American Central Insurance Company*;^39^ the law on the revocability of authority; ratification; and other cases on the conferral of powers more generally.

### A. Powers of Attorney

Powers of attorney provide the best evidence that agent consent is not a precondition for the conferral of authority under English law. Despite having been recognised by the common law for many decades, powers of attorney are poorly understood. A power of attorney is ‘a formal instrument by which one person empowers another to represent him, or act in his stead for certain purposes’.^40^ A power of attorney ‘is in principle no more than the grant of a form of agency’.^41^ ‘The position of a donee of a power of attorney is merely to act as agent for the principal.’^42^

Powers of attorney today are generally executed as deeds,^43^ specifically, deeds poll.^44^ A deed poll constitutes a purely unilateral act by the person who executes it, unlike deeds *inter partes*, which are (at least) bilateral and are executed by the parties to the transaction.^45^ Execution of a power of attorney by deed poll at common law requires writing, sealing and delivery,^46^ but not registration^47^ or donee consent.^48^ Even ‘delivery’ does not require acceptance by the donee, because ‘delivery’ does not require physical delivery of the deed.^49^ As *Vincent v Premo Enterprises (Voucher Sales)* explains:

A deed is binding on the maker of it, even though the parts have not been exchanged, as long as it has been signed, sealed and delivered. ‘Delivery’ in this connection does not mean ‘handed over’ to the other side. It means delivered in the old legal sense, namely, an act done so as to evince an intention to be bound. Even though the deed remains in the possession of the maker, or of his solicitor, he is bound by it if he has done some act evincing an intention to be bound, as by saying: ‘I deliver this my act and deed.’^50^

A principal can thus execute a power of attorney unilaterally, without the donee’s participation or acceptance. This view is confirmed by the case law.

In the leading case, *Abbott v UDC Finance Ltd*, the defendants were subscribers for shares in a private partnership for horse breeding.^51^ The claimant finance house had lent money to the partnership. The loan documents had been executed by the partnership’s promoter, who acted under powers of attorney signed by the defendants. The horse breeding market collapsed; the defendants’ investment failed. The claimant then demanded that the defendants repay the loan. In the summary judgment application, the defendants argued that the powers of attorney were invalid because they had never been accepted by the promoter and that any purported acceptance was ineffective as being contrary to a New Zealand statute.

The Court of Appeal of New Zealand rejected this argument, explaining that: ‘The giving of a power of attorney is a unilateral act. Its validity does not depend upon “acceptance” by the attorney, although the act of the attorney is necessary to exercise the authority which the power confers.’^52^

The power of attorney was thus valid, so that the loan between the claimant and defendants was valid. The defendants were required to repay the loan.

Other cases have likewise described powers of attorney as ‘one-sided’, not requiring the agent’s participation for their validity. In *Chatenay v Brazilian Submarine Telegraph Co Ltd*,^53^ Lindley LJ memorably described a power of attorney as a ‘one-sided instrument’, ‘an instrument which expresses the meaning of the person who makes it, but is not in any sense a contract’.^54^ This was material to the resolution of the case, which concerned the interpretation of the power of attorney. As a one-sided document, it was the meaning of the donor which was relevant in interpretating the power.^55^ Lindley LJ’s statements in *Chatenay* were followed in *Sinfra Akt v Sinfra Ltd*, which again described a power of attorney as a ‘one-sided instrument’.^56^ This characterisation was again material to the resolution of the dispute. As a one-sided instrument to which English law applied, the power of attorney was revocable at any time by the donor of the power.^57^ The donor’s purported revocation was thus effective.

In its Report on *The Incapacitated Principal*, the Law Commission accepted that, unlike statutory enduring powers of attorney in other jurisdictions which require the agent’s consent or acceptance for the enduring power to be valid, ‘This is, of course, not a requirement of an ordinary power of attorney in this country’.^58^ The Law Commission’s later work likewise assumes that ordinary powers of attorney are created unilaterally by the donor.^59^

In this respect, powers of attorney operate similarly to how deeds poll in other areas of law operate. Long-standing authority dating back to the 17th century shows that gifts can be validly made by an executed deed.^60^ The donee need not know of the deed or consent to the gift for the deed to be effective.^61^ As Ventris J said in 1690:

Conveyances at the common law do immediately (upon the execution of them on the grantor’s part) devest the estate out of him, and put it in the party to whom such conveyance is made, though in his absence, or without his notice, till some disagreement to such estate appears.^62^

A validly executed deed is thus sufficient to vest title to the donee.^63^ This analysis coheres with the view that powers of attorney, given by deed poll, likewise immediately confer authority on the agent.

Since powers of attorney are examples of agency, they provide the best evidence that authority can be conferred on an agent without the agent’s consent. ‘Powers of attorney are on general a good check on agency principles.’^64^ Unlike other controversial examples such as agency of necessity, powers of attorney can hardly be marginalised for being rare in practice. By 2020, over four million lasting powers of attorney had been registered under statute;^65^ instances of common law powers of attorney are likely to be considerable as well.

### B. Ruggles v American Central Insurance Company

Earlier, we saw that the outcome of some cases depends on which view is adopted. *Ruggles v American Central Insurance Company*,^66^ a rare, reported case of this type, suggests that agent consent is not a necessary condition for authority to be conferred.^67^

In *Ruggles*, an insurance company wanted to appoint Sedgwick as its agent. The company, through its general agent, initially conferred a limited authority on Sedgwick under which Sedgwick did not have authority to insure ‘special risks’. The company later sent a wider commission of authority which did not contain those restrictions to Sedgwick by post. The commission of authority was sent on 13 October, but it was only received by Sedgwick on 20 October. On 16 October, Sedgwick purported to insure a ‘special risk’ in favour of the claimants which put the company on risk from that day, and the risk eventuated on 19 October.

The New York Court of Appeals held that the company was bound even though the risk was a ‘special risk’. The main ground for their decision was what we would analyse today as apparent authority: a separate letter sent by the company secretary on the 13 October represented that Sedgwick was the company’s general agent; this letter had been shown to the claimants on 16 October and, on its face, it did not contain any apparent limitations of Sedgwick’s authority to act as general agent.^68^

However, an alternative ground for the court’s decision is actual authority. The analysis is as follows. Sedgwick initially had a limited actual authority, but this authority was expanded by the commission of authority sent by post. Since the policy was entered before the commission of authority was received, this suggests that the wider authority had been effectively conferred on Sedgwick without its consent. If so, then the agent’s consent is not necessary for the conferral of authority.

*Ruggles* is taken as a key case supporting this view of actual authority by an important text, the US *Restatement (Third)*, which regards it as a case showing that the agent can have actual authority even where the agent is unaware of the principal’s manifestation of intention that the agent act for him.^69^ If so, the agent’s consent cannot be a necessary condition for authority to be conferred on him. Other English writers similarly regard *Ruggles* as a key authority showing that the agent’s consent is unnecessary for authority to be conferred on him.^70^

### C. Revocation

A third piece of evidence is the law on the termination of authority. If authority is conferred for a reason, then, when that reason ends, authority should likewise terminate. Examining when authority terminates can thus shed light on when and why authority is conferred.

It is well established that authority is generally revocable at any time by the principal, even if doing so constitutes a breach of contract between principal and agent.^71^ This point is sometimes taken as proof that the agent’s consent is not necessary for authority to be conferred.^72^ But this view is not quite correct; it only shows that the *principal’s* consent is necessary for the conferral of authority. As the principal’s consent was necessary for authority to be granted to the agent, then, when the principal withdraws that consent, authority ends.

The true point is more subtle. What is important is that authority is generally revocable *only* by the principal. There is no suggestion that the *agent* alone can revoke authority that it has been granted. Indeed, the leading text suggests that the agent’s authority only ends if the agent’s ‘renunciation’ is accepted by the principal.^73^ This is easily explained on the basis that the agent’s consent is not a necessary condition for authority to be conferred on him. Since it is not, the agent’s subsequent withdrawal of consent cannot bring that authority to an end.

### D. Ratification

Ratification also suggests that authority can be conferred on an agent without the agent’s consent.^74^ Subject to certain prerequisites, principals can ratify unauthorised acts done on their behalf by others.^75^ Ratification operates to confer authority on the agent retrospectively, thus rendering previously unauthorised acts retrospectively authorised.^76^ It is ‘equivalent to an antecedent authority’.^77^

Like the conferral of authority by powers of attorney, ratification also appears to require only a ‘unilateral manifestation of intent’.^78^ Thus, a principal can ratify an unauthorised act done by the agent on behalf of the principal with a third party, T, without communicating the ratification to T. Some statements go even further, suggesting that there is no need to communicate ratification to the agent. In *Harrisons & Crossfield Ltd v London and North-Western Railway Company*, Rowlatt J held that:

Now, ratification does not rest upon estoppel. It need not be communicated to the party alleging it. Ratification is a unilateral act of the will, namely, the approval after the event of the assumption of an authority which did not exist at the time. It may be expressed in words or implied from or involved in acts.^79^

Likewise, in *Brown v Innovatorone plc*,^80^ Hamblen J said that ‘Ratification follows from the nature of the act and may even be a private act. There is no requirement of communication’.^81^

If ratification—a retrospective conferral of authority—need not even be communicated to the agent to be effective, then this indicates that authority can be conferred without the agent’s consent.

### E. Conferral of Powers Elsewhere

The law on the conferral of powers outside agency also suggests that it is possible for powers to be conferred unilaterally. The best examples involve trusts. In *In Re Byron’s Settlement*,^82^*In Re Park*^83^ and *In Re Jones*,^84^ the settlor conferred on a life interest on a beneficiary, and directed that after the beneficiary’s death, the fund should be held on trust for persons as the beneficiary should appoint. In all three cases, the court held that by the latter direction, the settlor gave the life beneficiary a valid power of appointment which could be exercised by the beneficiary. In all cases, there was no objection that the beneficiary had not consented to the conferral of the power. Though outside agency, these examples suggest that powers can be conferred unilaterally. Actual authority, a power, ought to be no different.

### F. Potential Objections

Defenders of mutual consent accounts might argue that some of these cases can be explained as ones where the agent consents by exercising the power. If so, then authority would only be conferred from the time where the agent purports to exercise it, but we could maintain that the conferral of authority requires the consent of both principal and agent.

However, this analysis is inconsistent with the case law, which indicates that powers of attorney validly confer authority once they are executed, not when the power was exercised. Nor can this analysis explain cases like *Notices*, discussed earlier.^85^ Where the agent does not exercise the power, such as in cases where notifications may be sent to the agent at his registered address or by email, or the agent simply acquires knowledge about matters falling within the scope of the conferred authority, it is difficult to find any act that manifests the agent’s consent to act for the principal.

These five pieces of evidence suggest that, contrary to some important views, authority can be conferred on agents without the agent’s consent.

## 5. The Agent’s Duties

Although a principal can confer authority on an agent unilaterally, that conferral of authority does not necessarily bring with it all the aspects of a fully-fledged agency relationship. While the external aspect of agency is present, the internal principal–agent relationship has two unusual features. In this section, I explore the first, arguing that while the principal can unilaterally confer authority on the agent, the principal cannot unilaterally impose agency-specific *duties* on the agent. For such duties to be owed by the agent, the agent must consent to act for the principal. This avoids an objection that the agent may be subject to, and breach, onerous duties without having consented to the agency.

### A. The Concern

One plausible reason for the popularity of ‘mutual consent’ accounts is the concern that the agent may be subject to onerous duties without having consented to acting for the principal. This concern was powerfully expressed by Seavey, who forcefully argued:

That the [agency] relationship is consensual there can be no doubt. The law creates the power upon the voluntary act of the principal and he is the dominus during its existence … *On the other side, the duties of a fiduciary cannot be thrust upon an unwilling person, so that the relation cannot be created, nor can it continue to exist without the consent of both parties*.^86^

Two examples illustrate:

*House Deadline*: P executes a power of attorney in favour of A, giving A power to sell P’s house. The power of attorney provides that A is obliged to sell the house within six months of the date of the execution of the power. P delivers the power of attorney to A’s registered address.

*Hungry Child*: P executes a power of attorney in favour of A, giving A power to use P’s assets to maintain P’s child. P delivers the power of attorney to A’s registered address. A has moved overseas for work and is unaware of the existence of the power of attorney.

In both cases, it would be onerous if A owed duties to sell the house or take reasonable care to maintain P’s child; he could be in breach of those duties for not acting when A did not consent to act as agent. In the second case, it would be even more onerous for A to owe duties where he did not even know of the existence of the power of attorney.

This concern is legitimate, but it does not arise under English law. To see why, it is helpful to look at a few different categories of agent duties. One caveat is in order: the topic of an agent’s duties is difficult, spanning the laws of contract, tort, restitution, fiduciary law and statute. They are not easy to categorise and may vary considerably: an estate agent is unlikely to owe exactly the same duties as a solicitor.^87^ All this makes generalisation about agent duties difficult.^88^ In what follows, I do not seek to provide an exhaustive exposition of all of an agent’s possible duties or when and why such duties are owed. My aim is more modest: to show how the agent who has authority conferred on him without his consent will not owe agency-specific duties.

### B. Positive Duties to Exercise the Authority

An obvious problem arises if the agent owes positive duties to exercise that authority, for instance in certain ways or within certain times, where he did not consent to act for the principal. But this situation does not arise because, under English law, an agent who does not consent to act for the principal owes the principal no duties to exercise the authority.

The best evidence is provided by powers of attorney. The standard position, stated in *R v Holt*, is that: ‘In so far as a power of attorney confers authority on the donee as agent of the donor, it operates merely as an authority to act and not as a direction to act.’^89^ Thus, a power of attorney confers ‘a mere bare authority to act which of itself imposes no obligation to act on the donee’.^90^

The Law Commission clearly explained the point in its Report, *The Incapacitated Principal*:

A power of attorney is not in itself a contract between donor and attorney. Accordingly, there is nothing about a power of attorney in the absence of any express provision or arrangement to the contrary that obliges the attorney to take any action under it. It just authorises him to do so if he wishes. He will, however, assume certain duties (including a duty of care) once he does start acting under the power.^91^

Powers of attorney thus confer authority on the agent but impose no duty on the agent to exercise it. In the absence of either a contract or an assumption of responsibility, the agent will generally owe no positive duties to act for the principal.^92^ Nor will the agent owe positive duties to exercise the authority within its scope, at particular times or in particular ways (eg with reasonable care) until and unless the agent agrees to act or assumes responsibility for so acting, or, possibly, when the agent in fact starts acting under the authority.^93^

An analogy can be drawn here with the conferral of dispositive powers in trusts. A settlor can settle property on trust, conferring dispositive powers^94^ on another. Conferral of these powers, like the conferral of authority, is a unilateral act, not requiring the donee’s consent for their validity.^95^ The donee of a mere non-fiduciary power owes no duty to exercise the power.^96^ Indeed, he does not even owe a duty to consider whether he ought to exercise the power.^97^ If, however, the non-fiduciary power holder exercises the power, various constraints apply.^98^

These examples show that powers can be conferred on an agent without the agent coming under duties to exercise them. The two need not coincide. Where powers are conferred unilaterally on a donee, the donee owes no duty to exercise the power at all or in certain ways (eg within a certain time) unless he agrees to do so, assumes responsibility for so doing or in fact starts acting under the power.

### C. Fiduciary Duties

More challenging are fiduciary duties, the subject of Seavey’s worries. Agents usually owe a range of duties falling under the ‘fiduciary’ label,^99^ including duties to act in the interests of the principal, to act in good faith, to avoid conflicts of interest with the principal and to not make unauthorised profits within the scope of the agency.^100^ This list is not free from controversy.^101^ There has been a vast literature on fiduciary law for decades, with a variety of accounts on offer. This, however, is not the place to discuss their relative merits. My point here is limited to showing that, according to some important cases and accounts of fiduciary law, the fiduciary’s consent to undertake his role or fiduciary duties is key to whether the fiduciary owes those duties. Where the agent has not consented to acting for the principal, he ought not owe fiduciary duties.

Important cases often indicate that fiduciary duties are owed where the fiduciary has undertaken to act for or on behalf of another in certain circumstances. In the leading case of *Bristol & West Building Society v Mothew*, Millett LJ explained that ‘A fiduciary is someone who has undertaken to act for or on behalf of another in a particular matter in circumstances which gives rise to a relationship of trust and confidence’.^102^ Here, Millett LJ was unmistakably influenced by Professor Paul Finn’s seminal book, *Fiduciary Obligations*,^103^ which is cited later in the same paragraph. Finn explained that:

For a person to be fiduciary he must first and foremost have bound himself in some way to protect and/or to advance the interests of another. This is perhaps the most obvious of the characteristics of the fiduciary office for *Equity will only oblige a person to act in what he believes to be another’s interests if he himself has assumed a position which requires him to act for or on behalf of the other in some particular matter*.^104^

This starting point has rarely been doubted. Over time, other scholars have sought to build on these insights in explaining when and why fiduciary duties arise.

Professor James Edelman, now a Justice of the High Court of Australia, argued that fiduciary duties are duties based on manifestations of a voluntary undertaking to another.^105^ They can therefore be located by looking at the terms expressed or implied in the voluntary undertaking. This account centres around the fiduciary’s voluntary undertaking to another. Where that undertaking is missing—where, for example, the agent has not consented to act for the principal—he ought not owe any fiduciary duties.

A different account was advanced by Lionel Smith, who regards the fiduciary relationship, rather than individual duties, as the key focal point for fiduciary law.^106^ He argues that most fiduciary relationships are voluntarily assumed.^107^ Voluntary assumption is again important here, but while ‘one must undertake the role… one is not required to undertake, individually, each of the obligations and incidents that the law attaches to the role’.^108^ Where that consent to undertake the role is absent, the agent usually ought not owe any fiduciary duties.

In a similar vein, the *Restatement (Third) of Agency* accepts that the agency relationship is fiduciary because ‘An agent *assents to act* subject to the principal’s control and on the principal’s behalf’.^109^ Where that assent is missing, the relationship ought not be fiduciary.

These accounts suggest that, for the agent to owe fiduciary duties to the principal, the agent must consent to act for the principal. It is difficult to find case law directly on point, but the existing evidence is consistent with the analysis offered here. No case imposes liability for breach of fiduciary duty in cases where authority is conferred unilaterally by the principal, and the agent has neither consented to acting for the principal nor actually started so acting. On the other hand, if the authority is exercised and misused, then the agent will be acting in breach of fiduciary duty.^110^

### D. Non-role-based Duties

This does not mean an agent who has authority conferred on him owes no duties at all to the principal unless he consents to act for the principal, just that he owes no *agency-specific* duties.

Consider the case where a principal confers written authority on an agent to sell the principal’s car. The agent owes duties to the principal not to hit him, not to make misrepresentations to him fraudulently^111^ and not to intentionally destroy the car by setting it on fire.^112^ However, these duties do not arise from the conferral of authority. Even if the agent had no authority, he would still owe these duties. This is because all persons in the world owe those duties, regardless of whether they have authority. Another such duty might be an agent’s duty to make restitution of a mistaken payment made to it by the principal.^113^ By contrast, the other duties discussed above, like duties to exercise the authority and possibly fiduciary duties, only seem to arise because the agent has been conferred the authority to act for the principal.^114^

It is thus necessary to distinguish between duties which an agent owes because authority is conferred on him and duties that are owed regardless of whether the agent has authority to act for the principal. For the former category, the agent only comes under duties where he consents to act for the principal. He will, however, owe duties in the latter category regardless of whether he consents. Duties in the latter category are not owed by virtue of the conferral of authority; the agent’s consent or lack thereof to the conferral is generally irrelevant to why the agent owes these duties.

## 6. The Option to Disclaim

When authority is conferred unilaterally on an agent, the internal relationship between principal and agent has another unique feature. This feature is linked to another potential objection: even if the agent does not owe onerous duties *qua* agent, he may not wish to have authority conferred on him at all. Why should he be forced to be an agent against his will?

English law has long since accepted the force of this objection in the context of unilateral gifts. Its traditional response is to accept that the gift is valid, but to then give the donee a choice: he can accept the gift or disclaim it *ab initio*. This approach should apply equally to the conferral of authority. The justification for giving the agent this choice is simple: no one should be compelled to be an agent against his will. Thus, another implication of showing that authority can be unilaterally conferred by the principal is the recognition of the agent’s ability to disclaim—a new ability that has not been recognised thus far.

### A. No One Should Be Compelled to Be an Agent against His Will

A simple and powerful idea may explain why agent consent has been thought to be central to the conferral of authority: that no one should be compelled to be an agent against his will. Behind this idea is the value of personal autonomy. One should be the author of one’s own life. This involves being free to choose the relationships one wishes to be involved in, on one’s own terms. In Kantian terms, ‘this idea of consent is an expression of each person’s entitlement to be his or her own master’.^115^ The prospective agent’s freedom of choice is lost if his consent to the agent’s authority is entirely irrelevant. He would be forced to take up a role he may not wish to have, which he is incapable of bearing or which he would choose only under certain terms.

Similar concerns have been recognised in the context of trustees and gift recipients. Some trusts and gifts can be constituted by the unilateral act of the settlor and donor respectively. There, it is well established that no one should be forced to be a trustee; a person ‘cannot be compelled to accept the burdens of … trusteeship against his or her will’.^116^ Likewise, gifts are not to be foisted on another against their will: ‘You certainly cannot make a man accept as a gift that which he does not desire to possess.’^117^ ‘The law certainly is not so absurd as to force a man to take an estate against his will.’^118^ In *Hardoon v Belilios*, the Privy Council recognised that ‘No one can be made the beneficial owner of [property] against his will’.^119^

### B. The Possibility of Disclaimer

In trusts and unilateral gifts, the law responds to these concerns by giving the trustee or donee who has not yet consented to the trust or gift a choice: he can accept or he can disclaim. The agent should have the same choice.

It is well established that the intended trustee ‘may choose whether he will accept the trust, or not’.^120^ In unilateral gifts where the donee’s consent is not a necessary condition for the passing of title,^121^ the same is true.^122^ In *Standing v Bowring*, shares were transferred into the joint names of the donor and her godson under a statute which did not require the donee’s consent for the transfer to be valid. Cotton LJ explained that the gift to the donees was valid, though subject to the donees’ ability to disclaim:

Now, I take the rule of law to be that where there is a transfer of property to a person, even although it carries with it some obligations which may be onerous, it vests in him at once before he knows of the transfer, subject to his right when informed of it to say, if he pleases, ‘I will not take it.’ When informed of it he may repudiate it, but it vests in him until he so repudiates it.^123^

Donees of ‘one-sided’ gifts thus have the choice as to whether they accept or disclaim the gift.^124^ As the Privy Council said in *Hardoon v Belilios*, ‘Any attempt to make [someone a beneficial owner of property against his will] can be defeated by disclaimer’.^125^

A similar choice should be given to agents. While authority can be conferred on an agent even without the agent’s consent, no agent should be compelled to act as an agent. This concern can be addressed by giving the agent a choice between accepting the authority or disclaiming it.

Like the conferral of authority, the acceptance or disclaimer of authority appear to be unilateral acts. Like trustees,^126^ an agent who wishes to accept can do so expressly, for example by acts conveying acceptance to the principal, or impliedly, for example by acting in connection with the agency.^127^ Likewise, disclaimer can be effected expressly or impliedly.^128^

### C. Disclaimer’s Retrospective Operation

In trusts, disclaimer operates retrospectively so that the intended trustee is treated as never having been a trustee at all.^129^ Gifts made by deed, which do not require physical delivery, operate likewise. Disclaimer of gifts thus renders the gift void *ab initio*, rather than operating as a re-transfer or re-conveyance of the subject matter of the gift: ‘the donor’s act alone is effective to transfer property to the donee, but, if the donee dissents, the gift is cancelled’.^130^ As the point was put in *Re Paradise Motor Co Ltd*, ‘a disclaimer operates by way of avoidance and not by way of disposition’.^131^ In *Sembaliuk v Sembaliuk*, it was said that ‘There is no donee of a disclaimed [testamentary] gift in a real sense. The bequest lapses … [A] disclaimer, being an avoidance of a gift, is not a conveyance of the property comprised in that gift.’^132^

Disclaimer’s retrospective operation means that if tax is charged on the subject matter of the gift during the period between the conferral of the gift and the gift being disclaimed, the donee of the gift is not liable for the tax if he disclaims.^133^ After disclaiming, the donee is treated as if title had never passed to him. The same is true if, for example, shares that are not fully paid up are settled on trust. If the beneficiaries of the trust disclaim, then they are not liable to indemnify the trustees on calls on the shares made between the settlement of the trust and the disclaimer.^134^

The disclaimer of authority should operate similarly. Perhaps surprisingly, there is little direct evidence in the agency context that suggests that retrospective disclaimer is possible. However, whether the agent can retrospectively disclaim so that he was never an agent may be important. Whether the agent has authority in the intervening period between conferral and disclaimer may sometimes be relevant for insurance, or possibly tax, purposes. It may also affect whether that authority falls within the control of the trustee-in-bankruptcy or liquidator if the agent commits an act of bankruptcy or insolvency in the intervening period but disclaims before an order of bankruptcy or winding-up procedure is entered.^135^ The only way to ensure that the agent neither gains benefits nor suffers disadvantages from having authority conferred on him is to enable him to disclaim *ab initio*. Since the disclaimer of trusts and gifts results in the apparent trustee or donee never being trustee or donee at all, disclaimer is no longer possible once the trustee has agreed to act as trustee^136^ or the donee has accepted the gift.^137^ The same should be true of agency.

## 7. Clarifying the Scope of ‘Mutual Consent’

The most significant implication of the argument advanced in this article is that it helps clarify the scope of what has long been accepted as the normative justification underpinning much of agency: mutual consent. Although courts and important writers alike support the view that mutual consent is at the heart of agency, such statements are insufficiently precise. The argument here illuminates one of the ways in which they are insufficiently precise: mutual consent is not at the heart of the conferral of authority. Authority can be conferred on the agent by a unilateral act of the principal.^138^ However, mutual consent may still be at the heart of other incidents of agency.

It is first necessary to separate the different conceptually distinct incidents of agency relationships. Not all incidents of agency arise at the same time or for the same reasons. Many such incidents may exist, for example the rights of agents to remuneration from their principals, agents’ warranty of authority to third parties or agents’ rights to recover expenses incurred in performing the agency. However, for the purposes of this article, three incidents are most important: the conferral of actual authority on the agent; the duties owed by an agent to the principal; and the creation of legal relations between principal and third party through the agent’s exercise of the actual authority he has been granted.

As this article has shown, in respect of the conferral of authority, statements about mutual consent’s centrality to agency are inaccurate.^139^ Authority can be conferred unilaterally by the principal, as powers of attorney and other examples indicate. There is no need for the agent to consent to the conferral by accepting the authority or agreeing to it for the authority to be validly conferred on the agent. Thus, mutual consent is not the normative reason justifying the conferral of authority. Instead, the unilateral conferral of authority is perhaps best justified on the basis of a version of the ‘will theory’.

However, this does not mean that statements about the centrality of mutual consent to agency must be completely rejected. Although inaccurate in relation to the conferral of authority, such statements are still accurate in relation to: (i) the duties between principal and agent; and (ii) the creation of legal relations between principal and third party through the agent’s exercise of authority.

As we have already seen, unlike the conferral of authority, the agency-specific duties owed by agent to principal only arise where the principal consents to the agent acting for him, and the agent consents to act for the principal. Mutual consent here plays an important role in justifying the creation of a special and distinctive relationship between principal and agent, one in which special duties are owed by agent to principal.

Mutual consent also seems important in justifying the legal relations created between principal and third party by the agent’s *exercise* of his authority. Where the principal grants authority to the agent, that authority is validly conferred, but need not be exercised by the agent. But if the agent exercises the actual authority conferred on him, effecting a change in the principal’s legal relations with the third party, for example, binding the principal to valid contractual rights and duties against the third party, the agent manifests his willingness to act for the principal, albeit that this manifestation is made to the third party. Again, the mutual consent of principal and agent play an important role in justifying the change of the principal’s legal relations through the agent’s exercise of his authority: the willingness of both that the agent can so act for the principal enables the principal to ‘multiply his legal personality in space’.^140^

Once suitably clarified, it is unnecessary to entirely jettison the view that mutual consent is at the heart of agency, as long as we are more precise about what we mean by ‘agency’. Such statements are meaningful, and do not mislead, when we are discussing either: (i) one part of the internal aspect of agency, the duties owed between principal and agent: or (ii) the external aspect of agency, the relationship created between the principal and a third party through the agent’s authorised acts. However, they are inaccurate if used to refer to the conferral of authority.

## 8. Conclusion and Implications

It is nearly universally accepted by courts and academic commentators that ‘mutual consent is at the heart of agency law’. Such statements, however, are too broad, requiring further unpacking and clarification. One plausible view is that such statements are claims about the conferral of authority.

In this article, I have sought to show that this view of the conferral of authority is incorrect. The agent’s consent is not a necessary condition for authority to be conferred on the agent. Instead, authority can be conferred unilaterally by the principal, for example through a written authority sent by post. However, when the principal does so, the relationship thereby formed between principal and agent has two unique features. First, the agent owes no agency-specific duties until and unless he manifests consents to act for the principal, and second, the agent has the option of accepting or retrospectively disclaiming the authority. The internal (principal–agent) aspect of agency is thus modified from that of the full agency relationship, though the external (principal–third party) aspect of agency remains unaffected.

The account advanced here helps clarify the scope of statements about mutual consent’s centrality to agency law. Currently, such statements are too wide. They cannot bear the weight that they are given. However, once suitably clarified, such statements need not be rejected entirely. Although mutual consent is not at the heart of the conferral of authority, mutual consent can meaningfully be said to be at the heart of other incidents of agency, such as the agent’s duties to the principal or the legal relations created between principal and third parties where the agent exercises his authority.

Two further implications of this account follow.

First, an advantage of this account of the conferral of authority is that it shows that English law is not an outlier on the international plane. The UNIDROIT Principles of International Commercial Contracts,^141^ the Principles of European Contract Law^142^ and the Draft Common Frame of Reference,^143^ all influenced to varying degrees by civilian jurisdictions, uniformly accept that the agent’s acceptance or consent to the authorisation is unnecessary for authority to be conferred.^144^ The German Civil Code provides likewise.^145^ On this point, English law is thought to be an outlier.^146^ This view is mistaken. Like these other approaches, English law does not require the agent’s consent for authority to be conferred on him. The English law of agency—and probably the common law of agency more generally—is not so very unique, but this is no bad thing.

Second, and more broadly, although this point cannot be definitively established here, this account suggests that the conditions for conferring powers on another differ from those for conferring rights on another. It appears that powers can generally be conferred unilaterally by the donor; the law seeks to give effect to the will of the power conferrer. Outside agency, in the law of trusts and wills, a settlor can unilaterally confer powers (of appointment, to add to a class of objects, to consent to certain decisions, etc) on another without the need for the latter’s consent. This is perhaps not an altogether surprising conclusion. To give someone a power, as long as that person is not duty bound to exercise that power, is to give them an *option*. Giving another an option is, generally, within one’s own control, and it is not ordinarily burdensome on the recipient. By contrast, conferring rights on another often requires the recipient’s consent, as gifts show. While a donor can make unilateral gifts through deeds, assignment, testamentary dispositions or under certain statutes, most cases of simple gifts, for example of chattels, will require the recipient to manifest consent or acceptance of the gift in some way. Having rights goes beyond merely having an option: rights may be beneficial, but they may also impose burdens in ways that holding unconstrained powers are not, for example requiring the recipient to pay associated taxes (eg property taxes). In relation to the conferral of authority, agency law may have more in common with the law of trusts and gifts than currently thought.

## Footnotes

1
*Garnac Grain Co v Faure & Fairclough Ltd* [1968] AC 1130 (HL) 1137; *Kelly v Cooper* [1993] AC 205 (PC) 213; *Alwie Handoyo v Tjong Very Sumito* [2013] 4 SLR 308, [148].

2 Peter Watts and FMB Reynolds, *Bowstead & Reynolds on Agency* (23rd edn, Sweet & Maxwell 2023) para 1-001 (*Bowstead & Reynolds*); Warren A Seavey, ‘The Rationale of Agency’ (1919) 29 Yale LJ 859, 863–4; Gerard McMeel, ‘Philosophical Foundations of the Law of Agency’ (2000) 116 LQR 387; Thomas Krebs, ‘Agency Law for Muggles: Why There Is No Magic in Agency’ in Andrew Burrows and Edwin Peel (eds), *Contract Formation and Parties* (OUP 2010); Roderick Munday, *Agency: Law and Principles* (4th edn, OUP 2022) para 1.24; Gino Dal Pont, *Law of Agency* (4th edn, LexisNexis 2020) paras 1.4, 4.1, 4.3, 4.4; American Law Institute, *Restatement (Third) of Agency* (2006) § 1.01; see also § 2.02.

3 Which may be language more familiar to civilian lawyers, see eg Nils Jansen and Reinhard Zimmermann (eds), *Commentaries on European Contract Laws* (OUP 2018) 610–12. The language of ‘grant’ is also used in the Principles of European Contract Law (PECL), Art 3:201; the UNIDROIT Principles of International Commercial Contracts (PICC), Art 2.2.2; the Draft Common Frame of Reference (DCFR), II 6:104.

4 This is a concept distinct from any contract between principal and agent: Michele Graziadei, Ugo Mattei and Lionel Smith, ‘A Short Note on Terminology’ in Michele Graziadei, Ugo Mattei and Lionel Smith (eds), *Commercial Trusts in European Private Law* (CUP 2005).

5 Such consent must be objectively manifested, but for brevity I refer simply to ‘consent’ throughout the rest of the article.

6 114 NY 415, 21 NE 1000 (1889).

7
*Bowstead & Reynolds* (n 2) para 1-001 (emphasis added).

8 Seavey (n 2) 868; see also 863.

9 ibid 863–4.

10
*Restatement (Third) of Agency*, § 1.01.

11 Roderick Munday, *Agency: Law and Principles* (4th edn, OUP 2022) para 1.24.

12 eg *Bowstead & Reynolds* (n 2) paras 4-007–4-008 (agency of necessity); McMeel (n 2) 409–10.

13 eg agency of necessity: *Bowstead & Reynolds* (n 2) para 4-008.

14 Dal Pont (n 2) 81; McMeel (n 2) 410.

15
*Bowstead & Reynolds* (n 2) para 1-004.

16
*G (A) v G (T)* [1970] 2 QB 643 (CA) 652 (Lord Denning MR) (minor as principal); *Re D’Angibau* (1880) 15 Ch D 228 (CA) 246 (Brett LJ) (minor as agent).

17
*Walsh v Whitcomb* (1797) 2 Esp 565; *Frith v Frith* [1906] AC 254; *Angove Pty Ltd v Bailey* [2016] UKSC 47, [2016] 1 WLR 3179.

18 It is important to distinguish the conferral of authority (a power) from the exercise of that power. The conferral, or grant, of authority by the principal gives the agent actual authority, which is a power to affect the principal’s legal relations. However, if unexercised in the agent’s hands, the principal’s legal relations remain unaffected. Unless the agent owes duties to exercise the authority or consider its exercise, whether the power is exercised is entirely up to the agent. An agent can have authority conferred on him but never exercise it.

19
*Bowstead & Reynolds* (n 2) para 1-004.

20 Howard Bennett, *Principles of the Law of Agency* (2nd edn, Hart Publishing 2024) 6.

21 Dal Pont (n 2) para 4.3.

22 ibid para 4.4.

23 [1968] AC 1130 (HL) 1137.

24
*Branwhite v Worcester Works Finance Ltd* [1969] 1 AC 552, 587; *Yasuda Fire & Marine Insurance Co of Europe Ltd v Orion Marine Insurance Underwriting Agency Ltd* [1995] QB 174, 185; *Yukong Line Ltd of Korea v Rendsburg Investments Corporation of Liberia and Ors (No 2)* [1998] 1 WLR 294, 303; *Magellan Spirit ApS v Vitol SA* [2016] 1 CLC 480, [18]; *Baylis v Commissioners for HMRC* [2016] UKFTT 0725 (TC) [124]-[128]; *Michael Tuke v JD Classics Ltd* [2018] EWHC 531 (QB) [35]; *Conway v Eze* [2018] EWHC 29 (Ch) [105].

25
*VMS Enterprises Ltd v The Brexit Party* [2021] SC GLA 49, [68].

26
*Leo Vincent Pola v Commonwealth Bank of Australia* [1997] FCA 1476.

27 [2013] 4 SLR 308, [148].

28 ibid [149].

29
*Michael Tuke v JD Classics Ltd* [2018] EWHC 531 (QB) [31]-[32]; *Scott v Davies* [2000] HCA 52, (2000) 204 CLR 333 [227] (Gummow J), citing *International Harvester Co of Australia Pty Ltd v Carrigan’s Hazeldene Pastoral Co* [1958] HCA 16, (1958) 100 CLR 644, 652; *UBS AG (London Branch) v Kommunale Wasserwerke Peipkiz GmbH* [2017] EWCA Civ 1567 [97]. cf apparent authority.

30 Wolfram Müller-Freienfels, ‘Legal Relations in the Law of Agency: Power of Agency and Commercial Certainty’ (1964) 13 Am J Comp L 193, 203 (emphasis added).

31
*Bowstead & Reynolds* (n 2) para 2-001.

32 ibid para 2-002.

33 ibid para 1-006.

34 ibid para 2-032.

35 [2023] EWHC 265 (Ch) [601].

36 Francis Reynolds and Cheng-Han Tan, ‘Agency Reasoning—a Formula or a Tool?’ [2018] Sing JLS 43.

37 See eg *Employers Liability Assurance Corp Ltd v Sedgewick Collins and Co Ltd* [1927] AC 95 (name of agent listed on company’s register); *Sino Channel Asia Ltd v Dana Shipping and Trading Pte Ltd* [2017] EWCA Civ 1703 (service of notice of arbitration).

38
*Tanham v Nicholson* (1872) LR 5 HL 561.

39 114 NY 415, 21 NE 1000 (1889).

40 Daniel Greenberg (ed), *Jowitt’s Dictionary of English Law* (5th edn, Sweet & Maxwell 2019).

41
*Gregory v Turner* [2003] EWCA Civ 183, [2003] 2 All ER 1114 [67] (Brooke LJ).

42
*Re Craven’s Estate* [1937] Ch 423 (Ch) 428 (Farwell J). See also *Crago v McIntyre* [1976] 1 NSWLR 729 (SC(NSW)) 749 (Holland J).

43 The Powers of Attorney Act 1971, s 1(1) requires that powers of attorney must be given under seal, though no such general requirement existed at common law. Today, powers of attorney given under seal are commonly assumed to be synonymous with powers of attorney executed as a deed: see eg *Re J (Enduring Power of Attorney)* [2009] EWHC 436 (Ch), [2010] 1 WLR 210 [4] (Lewison J); *Commissioner of Stamps (Vic) v Papalia* (1982) 12 ATR 866 (SC(Vic)) 869–70 (Crockett J); *Houston v Houston* (2012) 352 DLR (4th) 125 (CA(BC)) [26] (Newbury JA).

44 Berna Collier and Shannon Lindsay, *Powers of Attorney in Australia and New Zealand* (Federation Press 1992) 3; Trevor Aldridge, *Powers of Attorney* (7th edn, Longman 1988); Law Commission, *The Incapacitated Principal* (Law Com No 122, 1983) para 4.17.

45 Deeds are either deeds poll or deeds *inter partes*: Nicholas Seddon, *Seddon on Deeds* (2nd edn, Federation Press 2022) 18.

46
*Goddard’s Case* (1584) 2 Co Rep 4b, 76 ER 396. The common law requirements have since been varied by statute (eg Law of Property (Miscellaneous Provisions) Act 1989), and some, such as the requirement of sealing, have been watered down over time.

47 Registration of powers of attorney was once mandatory in very limited cases and otherwise voluntary (most recently under the Judicature Act 1925, s 219, with that provision being traceable to the Conveyancing Act 1881, s 48). These provisions were repealed in the Powers of Attorney Act 1971, as suggested by the Law Commission: Law Commission, *Powers of Attorney* (Law Com No 30, Cmnd 4473, 1970) paras 2–10.

48
*Barlow v Heneage* (1702) Prec Ch 211; *Clavering v Clavering* (1704) Prec Ch 235, 2 Ver 473; *Doe d Garnons v Knight* (1826) 5 B & C 671, 108 ER 250; *Exton v Scott* (1833) 6 Sim 31, 58 ER 507. Contrast Mental Capacity Act 2005, Sch 1, para 1-2 (lasting powers of attorney).

49
*Xenox v Wickham* (1867) LR 2 HL 296 (HL) 323 (Lord Cranworth). This was not always the law, see DEC Yale, ‘The Delivery of a Deed’ (1970) 28 CLJ 52.

50 [1969] 2 QB 609 (CA) 619 (Lord Denning MR).

51 [1991] NZCA 97, [1992] 1 NZLR 405.

52 ibid 414. See also *National Australia Finance Ltd v Fahey* [1990] 2 NZLR 482.

53 [1891] 1 QB 79 (CA).

54 ibid 85.

55 ibid.

56 [1939] 2 All ER 675 (KB) 682 (Lewis J).

57 ibid.

58 Law Commission, *The Incapacitated Principal* (Law Com CP No 69, 1976) para 71.

59 Law Com No 122 (n 44) paras 2.2–2.3.

60 Jonathan Hill, ‘The Role of the Donee’s Consent in the Law of Gift’ (2001) 117 LQR 127, 134. When a deed has been validly executed, it is effective to pass title to the donee; the gift is thus validly made at this point, subject to the donee’s disclaimer, as discussed later in section 6.

61 ibid 133–4.

62
*Thompson v Leach* (1690) 2 Vent 198, 201, upheld by the House of Lords.

63 Robert F Norton, Robert JA Morrison and Hugh J Goolden, *A Treatise on Deeds: Norton on Deeds* (2nd edn, Sweet & Maxwell 1928) 22–3. See later *Crewe v Dicken* (1798) 4 Ves 96; *Siggers v Evans* (1855) 5 E&B 367, 24 LJ QB 305; *Mallott v Wilson* [1903] 2 Ch 494; *Boughton v Boughton* (1739) 1 Atk 625; *Doe d Garnons v Knight* (n 48); *Macedo v Stroud* [1922] 2 AC 330 (PC).

64 Reynolds and Tan (n 36) 46.

65 Lasting powers of attorney do require the agent’s consent and registration, along with a host of other safeguards, because they operate only where the principal has lost capacity. Additional safeguards are necessary to protect the principal’s interests in such cases because the principal is not able to revoke the authority due to lack of capacity. Evidence is available from the Office of the Public Guardian <https://publicguardian.blog.gov.uk/2020/07/17/weve-launched-the-new-use-a-lasting-power-of-attorney-service/>.

66 114 NY 415, 21 NE 1000 (1889).

67 Although *Ruggles* is an American case, American and English law on agency were materially similar in the late 19th century when *Ruggles* was decided; both were simply thought to be illustrations of a common law of agency, see eg Deborah DeMott, ‘The Contours and Composition of Agency Doctrine: Perspectives from History and Theory on Inherent Agency Power’ (2014) 2014 U Ill L Rev 1813.

68 ibid 1002.

69 Though it disagrees with this result. See § 3.01 (creation of actual authority), Reporter’s Notes, point b; see also § 2.01 (actual authority).

70 eg Bennett (n 21) 6.

71
*Angove’s Pty Ltd v Bailey* [2016] UKSC 47, [2016] 1 WLR 3179.

72 Francis Reynolds, ‘Agency’ in Andrew Burrows (ed), *English Private Law* (2nd edn, OUP 2013), [9.06].

73
*Bowstead & Reynolds* (n 2) paras 10-002, 10-004, 10-023: any renunciation by the agent must be accepted by the principal for actual authority to be terminated.

74
*Bowstead & Reynolds* (n 2) para 2-078.

75
*Firth v Staines* [1897] 2 QB 70 (QB).

76
*Kelner v Baxter* (1866–67) LR 2 CP 174; *Bolton Partners v Lambert* (1889) 41 Ch D 295.

77
*Koenigsblatt v Sweet* [1923] 2 Ch 314 (CA) 325 (Lord Sterndale MR).

78 As described by PICC, Art 2.2.9.

79 [1917] 2 KB 755 (KB) 758 (emphasis added). Approved of in *Shell Co of Australia Ltd v Nat Shipping and Bagging Services Ltd (The Kilmun)* [1988] 2 Lloyd’s Rep 1 (CA).

80 [2012] EWHC 1321 (Comm).

81 ibid [862].

82 [1891] 3 Ch 474.

83 [1932] 1 Ch 580.

84 [1945] Ch 105.

85 See section 3D above.

86 Seavey (n 2) 863 (emphasis added).

87
*Bowstead & Reynolds* (n 2) para 6-001.

88
*Torre Asset Funding Ltd v Royal Bank of Scotland plc* [2013] EWHC 2670 (Ch) [147].

89 [1983] Vic SC 552 (19 December 1983).

90 Collier and Lindsay (n 44) 161.

91 Law Com No 122 (n 44) para 2.5.

92
*Bowstead & Reynolds* (n 2) paras 6-002–6-003.

93 In some cases, it may not necessarily be easy to tell whether the agent starts acting under the authority. For an illustration, see *Prebble & Co v West* (1969) 211 EG 831, 832 (Edmund Davies LJ).

94 eg powers of appointment: *Freme v Clement* (1881) 18 Ch D 499, 504; *Re Dilke* [1921] 1 Ch 34; *Re Manisty’s Settlement* [1974] Ch 17; *Re Hay’s Settlement Trusts* [1982] 1 WLR 202. See also unilateral contracts, where the agent is also under no duty to exercise the authority: *Luxor (Eastbourne) Ltd v Cooper* [1941] AC 108, 124 (Lord Russell) (unilateral commission contracts with estate agents); *Ryan v Pilkington* [1959] 1 WLR 403.

95 Implicit in Geraint Thomas, *Thomas on Powers* (2nd edn, OUP 2012), paras 3.02–3.14.

96
*Re Gulbenkian’s Settlements* [1970] AC 508, 518.

97
*Re Hay’s Settlement Trusts* [1982] 1 WLR 202, 209, 210; *McPhail v Doulton* [1971] AC 424, 449; *Gartside v IRC* [1968] AC 553.

98 He must do so within the scope of the power, and for proper purposes. See Thomas (n 95) chs 8–9; *Duke of Portland v Topham* (1864) 11 HLC 43, 54; *Vatcher v Paull* [1915] AC 372, 378.

99 eg *Restatement (Third) of Agency*, § 8.01, comment b.

100
*Bristol and West Building Society v Mothew* [1998] Ch 1, 18 (Millett LJ).

101 There has been a vast literature on fiduciary law for decades, with a variety of accounts on offer. Some prominent works include Paul Finn, *Fiduciary Obligations* (LawBook Co 1977); Matthew Conaglen, *Fiduciary Loyalty: Protecting the Due Performance of Non-Fiduciary Duties* (Hart Publishing 2010); Lionel Smith, *The Law of Loyalty* (OUP 2023). Why fiduciary duties, whether fiduciary relationships or fiduciary duties, are the key analytical focal point, what conduct is prescribed or proscribed by fiduciary law and other such questions cannot be definitively resolved here.

102
*Bristol and West* (n 100) 18.

103 Finn (n 101).

104 ibid, reprinted as Paul Finn, *Fiduciary Obligations: 40th Anniversary Republication with Additional Essays* (Federation Press 2016) 9–10 (emphasis added).

105 James Edelman, ‘When do Fiduciary Duties Arise?’ (2010) 126 LQR 302.

106 Lionel Smith, ‘Fiduciary Relationships: Ensuring the Loyal Exercise of Judgement on Behalf of Another’ (2014) 130 LQR 608; Lionel Smith, ‘Parenthood Is a Fiduciary Relationship’ (2020) 70 UTLJ 70.

107 Smith, *The Law of Loyalty* (n 101) 69–71.

108 ibid 70.

109
*Restatement (Third) of Agency*, § 8.01, comment b (emphasis added).

110
*The Margaret Mitchell* (1858) Swab 372, 166 ER 1174, though using the language of ‘breach of trust’ at a time when ‘fiduciary’ language was less well established.

111 Breach of which would be deceit, eg *Standard Chartered Bank v Pakistan National Shipping Corporation (Nos 2 and 4)* [2002] UKHL 43, [2003] 1 AC 959.

112 Breach of which would amount to at least trespass to goods, if not conversion, unless the agent fell within the exception identified by Blackburn J in *Hollins v Fowler* (1874–75) LR 7 HL 757, 767–8.

113
*Kelly v Solari* (1841) 9 M&W 54, 152 ER 24.

114 Statutory duties imposed on certain classes of agents probably also fall into this category, eg estate agents, see the Estate Agents Act 1979 (as amended by the Enterprise Act 2002); Consumers, Estate Agents and Redress Act 2007, Sch 6; Unfair Terms in Consumer Contracts Regulations 1999, applied in *Office of Fair Trading v Foxtons Ltd* [2009] EWCA Civ 288, [2010] 1 WLR 663.

115 Arthur Ripstein, *Force and Freedom* (Harvard UP 2009) 108.

116
*Scarpuzza v Scarpuzza* [2011] WASC 65 [47], citing *Thompson* (n 62) 20; 86 ER 391, 397.

117
*Standing v Bowring* (1885) 31 Ch D 282 (CA) 286 (Lord Halsbury).

118
*Townson v Tickell* (1819) 3 B & Al 31 (KB) (Abbott CJ) (testamentary gift).

119 [1901] AC 118 (PC) 123.

120
*Robinson v Pett* (1734) 3 Peere Wms 249, 251; 24 ER 1049, 1050.

121 Examples include the creation of trusts in favour of beneficiaries and gifts effected by deed.

122 See generally Hill (n 60).

123
*Standing* (n 17) 288 (Cotton LJ).

124
*Thompson* (n 62) 201 (Ventris J); *Hardoon v Belilios* [1901] AC 118 (PC) 123.

125 [1901] AC 118 (PC) 123.

126
*Jones v Higgins* (1866) LR 2 Eq 538; *Ong v Ping* [2015] EWHC 1742 (Ch) [98], point not considered on appeal: [2017] EWCA Civ 2069.

127 eg in trusts, where acceptance may be implied from the trustee accepting the trust property (*Ong v Ping* (n 126) [98]), joining in assigning a lease held on the trust (*Urch v Walker* (1838) 3 Myl & Cr 702, 40 ER 1097),or bringing an action on behalf of the trust (*Montford v Cadogan* (1810) 17 Ves Jr 485, 488–89; 34 ER 188, 189).

128 Express disclaimer in trusts: eg *Norway v Norway* (1834) 2 My & K 278, 39 ER 950; *Foster v Dawber* (1860) 1 Dr & Sm 172; *Re Birchall* (1889) 40 ChD 436; *Orlanski v Spiegel* [2015] VSC 662. Ideally by deed with a recital that the trustee has not acted in connection with the administration of the trust: Lynton Tucker, Nicholas Le Poidevin and James Brightwell (eds), *Lewin on Trusts* (20th edn, Sweet & Maxwell 2020) para 13-008 (*Lewin on Trusts*); *Re Schär* [1951] Ch 280, 284 (deed poll). Implied disclaimer in trusts can also be effected by acting in a way inconsistent with acceptance: *Lewin on Trusts*, paras 13-009–13-010.

129
*Doe d Chidgey v Harris* (1847) 16 M & W 517, 524; *Peppercorn v Wayman* (1852) 5 De G.

130 Hill (n 60) 128.

131 [1968] 1 WLR 1125, 1143 (Danckwerts LJ).

132 (1984) 15 DLR (4th) 303 (Alberta Court of Appeal) 309–10 (Stevenson JA).

133 Hill (n 60) 114.

134 eg *Hardoon* (n 124), though in that case the beneficiaries had accepted the trust.

135 The Insolvency Act 1986, s 283 vests the bankrupt’s estate in the trustee. The bankrupt’s estate does not include property ‘held … on trust for any person’ (s 283(3)(a)), but can include powers. See *Lewin on Trusts* (n 128) para 28-095.

136
*Re Sharman’s Will Trusts* [1942] Ch 311 (Ch) 314.

137 See *Bence v Gilpin* (1868) LR 3 Ex 76, 84 (Channell B).

138 Another source of imprecision concerns a different sense in which ‘mutual’ might be understood, in the sense of dependent or independent consents by principal and agent. By contrast, what I am referring to is whether the consents are bilateral or unilateral. It seems fairly clear that such consents, even if they are bilateral, need not be dependent ones. Thus, an agent may act, thinking she is overstepping her actual authority but hoping that the principal will ratify, when she has in fact had authority conferred on her by the principal even though that authority has not been communicated to her. Proponents of ‘mutual consent’ views would accept that there is ‘mutual consent’ in this scenario even though the consents of principal and agent are independent.

139 See section 4.

140 Percy H Winfield, *Pollock on Contract* (13th edn, Stevens & Sons 1950) 45; Rachel Leow, ‘Understanding Agency: A Proxy Power Definition’ (2019) 78 CLJ 99, 114–15.

141 PICC, Art 2.2.2(1).

142 PECL, Art 3:201(1).

143 DCFR, II 6:103.

144 Jansen and Zimmermann (n 3) 610.

145 Bürgeliches Gesetzbuch, § 167; see also the commentary by Hannes Wais in Gerhard Dannemann and Reiner Schulze (eds), *German Civil Code: Bürgeliches Gesetzbuch (BGB) Vol I* (CH Beck 2020) § 167, 1–6.

146 ibid 610, fn 4.

## Notes

I am very grateful to the two anonymous referees for their helpful comments, which enabled me to improve this article substantially. Earlier drafts of this article were presented to audiences at the LSE, Bristol and Toronto; I am very grateful to the organisers and all the attendees for their generous engagement. I am also grateful to Jason Neyers, Robert Stevens and Andreas Televantos for comments and assistance. Special thanks are owed to Francis Reynolds, who read several drafts at different stages of progress and provided many helpful interventions, saving me from error more than once. All remaining errors are mine.

